# Measurement Biases Explain Discrepancies between the Observed and Simulated Decadal Variability of Surface Incident Solar Radiation

**DOI:** 10.1038/srep06144

**Published:** 2014-08-21

**Authors:** Kaicun Wang

**Affiliations:** 1 State Key Laboratory of Earth Surface Processes and Resource Ecology, College of Global Change and Earth System Science, Beijing Normal University, Beijing, 100875, China

## Abstract

Observations have reported a widespread dimming of surface incident solar radiation (*R_s_*) from the 1950s to the 1980s and a brightening afterwards. However, none of the state-of-the-art earth system models, including those from the Coupled Model Intercomparison Project phase 5 (CMIP5), could successfully reproduce the dimming/brightening rates over China. We find that the decadal variability of observed *R_s_* may have important errors due to instrument sensitivity drifting and instrument replacement. While sunshine duration (SunDu), which is a robust measurement related to *R_s_*, is nearly free from these problems. We estimate *R_s_* from SunDu with a method calibrated by the observed *R_s_* at each station. SunDu-derived *R_s_* declined over China by −2.8 (with a 95% confidence interval of −1.9 to −3.7) W m^−2^ per decade from 1960 to 1989, while the observed *R_s_* declined by −8.5 (with a 95% confidence interval of −7.3 to −9.8) W m^−2^ per decade. The former trend was duplicated by some high-quality CMIP5 models, but none reproduced the latter trend.

Solar radiation is the ultimate energy source of the earth's climate system, which drives weather and climate change and vegetation growth^1^. Due to its importance, surface incident solar radiation (*R_s_*) has been widely measured since the late 1950s. These observations have been archived by the Global Energy Balance Archive (GEBA)^2^. They have suggested a widespread decrease between the 1950s and 1980s (“global dimming”)^2^ with the subsequent brightening^3^,^4^.

Recent studies^5^,^6^ have compared the Coupled Model Intercomparison Project phase 3 (CMIP3) and phase 5 (CMIP5)^7^ decadal variability of simulated *R_s_* with observations collected by GEBA over land for the last half century and have reported large discrepancies. In particular, it was found that all of the 42 models of CMIP5 underestimated the trend of the observed *R_s_* and none of the simulations of the CMIP5 models could reproduce the magnitude of the observed dimming trend over China and India^5^. From these comparisons, it could be inferred that the models are seriously flawed in their determination of the decadal variability of *R_s_*. If this is so, their reported good ability to reproduce twentieth century climate could be called into question because *R_s_* has an important contribution to decadal variability of surface air temperature^8^,^9^,^10^.

In this study, we examine the decadal variability of observed *R_s_* in China and find that the observed *R_s_* may have overestimated the dimming rate due to the degradation problem of instruments before 1990 and overestimated the brightening rate due to instrument replacement from 1990 to 1993. When these sources of error are accounted for, the inference that there is serious disagreement between models and observations becomes much weaker.

## Results

Here, we evaluate the decadal variability of the observed *R_s_* with an independent measurement of sunshine duration (SunDu). SunDu is a conventional meteorological observation that records the time duration during a day when the direct solar beam is greater than 120 W m^−2^, which is defined by the world meteorological organization. The light-sensitive paper of a SunDu recorder is burned when the direct solar beam is higher than a threshold, i.e., 120 W m^−2^. SunDu is measured through reading the length of the burned mark during a day.

*R_s_* can be calculated from SunDu, i.e., with the method (Eq. (1)) proposed by Yang et al.^11^, which has been regarded as having the capability of reflecting the impacts of clouds and aerosols on *R_s_* accurately^12^,^13^,^14^,^15^,^16^,^17^. To apply the method globally or regionally, existing studies^12^,^13^ tried to derive a suite of parameters by calibrating the equations with the observed *R_s_*.

**Figure 1:**
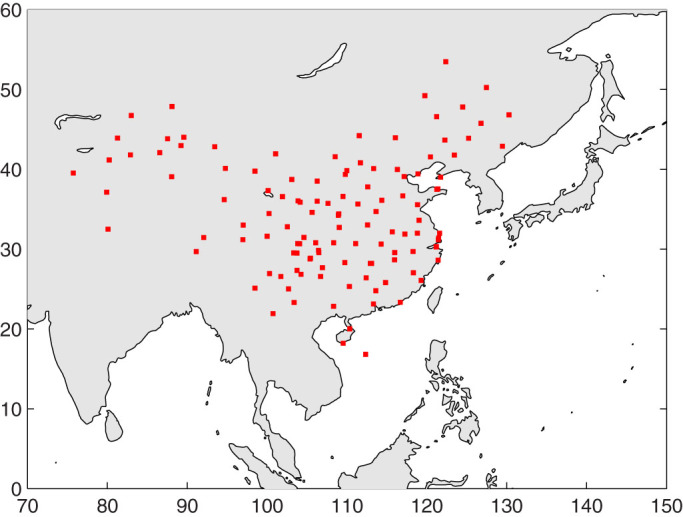
A map of the stations (red square) where data durations of both surface incident solar radiation (*R_s_*) and Sunshine Duration (SunDu) were longer than 120 months from 1958 to 2012. There are 122 stations in total. The figure was produced using MATLAB.

However, the burning threshold depends on the types of SunDu recorders and the sitting environment, i.e., its geographical location^16^. To address this issue, we calibrate the Eq. (1) to estimate *R_s_* from SunDu at each station. We find 122 stations where both *R_s_* and SunDu were measured for more than 10 years from 1958 to 2012 (Fig. 1), from which a reliable relationship between *R_s_* and SunDu can be established at each station (Eq. (1) of Method Section). The relationship between *R_s_* and SunDu may vary with geo-location and types of SunDu recorders. However, this relationship is expected to be stable for a station given the same SunDu recorder has been used. Under the assumption that the relationship between *R_s_* and SunDu is stable for a station, the calibrated equation can be used to calculate *R_s_* from SunDu for a longer time period, as SunDu has a longer observation history than *R_s_*^16^.

**Figure 2:**
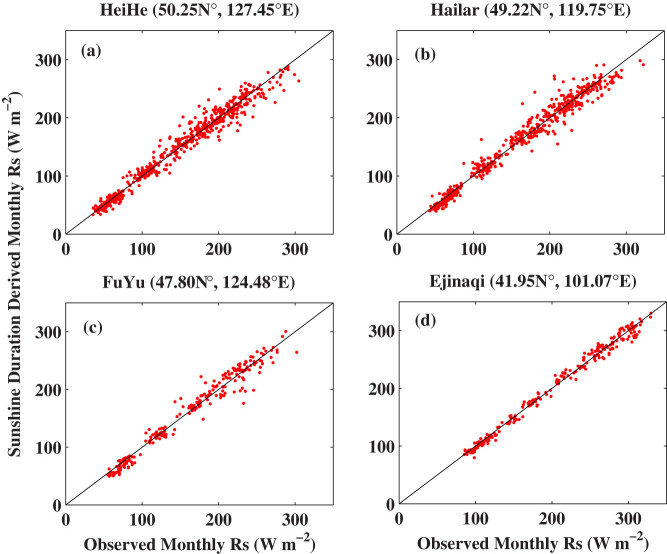
Scatterplots of monthly averages of Sunshine Duration (SunDu) derived surface incident solar radiation (*R_s_*) as a function of the observed *R_s_* at four stations. The biases, standard deviations, and correlation coefficients of the comparisons between SunDu-derived *R_s_* and observed *R_s_* at the four stations are: (a): −0.3 W m^−2^, 12.0 W m^−2^, 0.99, (b) −0.6 W m^−2^, 12.9 W m^−2^, 0.98, (c) −0.8 W m^−2^, 12.5 W m^−2^, 0.98, and (d) −0.4 W m^−2^, 7.9 W m^−2^, 0.99. The biases are near zero because Eq. (1) used to calculate *R_s_* from SunDu was calibrated at each station with the observed *R_s_*. Four examples are shown here and the statistical results of all the stations are shown in Fig. 3. The figure was produced using MATLAB.

**Figure 3:**
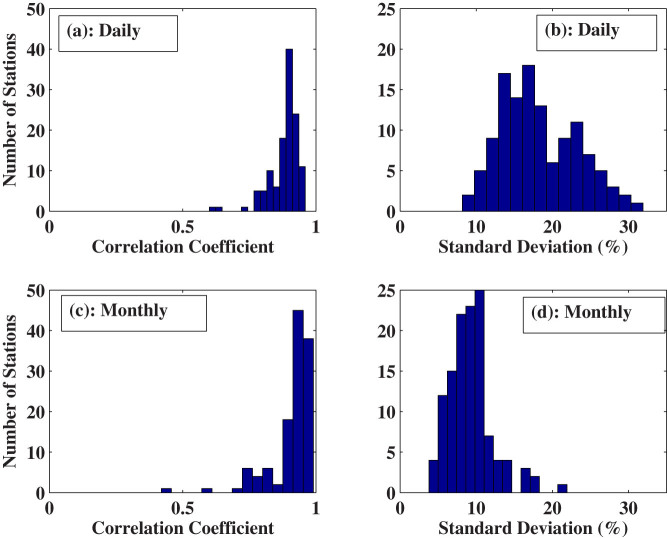
Histograms of the statistical parameters of the comparisons between daily and monthly observed surface incident solar radiation (*R_s_*) and *R_s_* derived from Sunshine Duration (SunDu). The correlation coefficients and relative standard deviations (normalized by multi-year averages at each station) calculated from daily data are shown in panels (a) and (b), and those calculated from monthly data are shown in panels (c) and (d). As the Eq. (1) used to calculate *R_s_* from SunDu was calibrated at each station, the biases of the comparisons are near zero and are not shown here. The medians of the correlation coefficients and the relative standard deviations between observed and calculated daily *R_s_* are 0.89 and 17.32%, respectively, and the values are 0.94 and 9.11% for monthly *R_s_*. The figure was produced using MATLAB.

Fig. 2 shows scatterplots of SunDu-derived *R_s_* as a function of the observed *R_s_* at four stations. The biases are near zero because Eq. (1) used to estimate *R_s_* from SunDu was calibrated with the observed *R_s_* at each station. The statistical results of the standard deviation and correlation coefficients of all the stations are shown in Fig. 3, which shows *R_s_* can be accurately estimated from SunDu at daily and monthly time scales. SunDu has difficulty in estimating *R_s_* at time scales shorter than one day, as only daily SunDu was documented.

**Figure 4:**
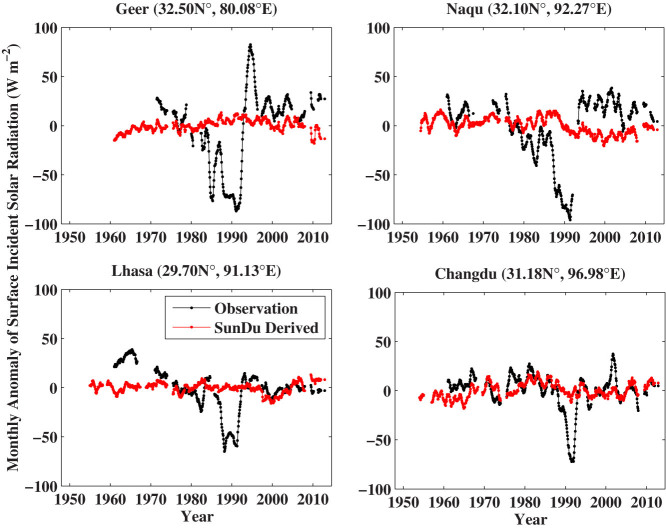
Time series of 12-month smoothed monthly anomalies of observed surface incident solar radiation (*R_s_*) and *R_s_* calculated from Sunshine Duration (SunDu) at four stations in the Tibetan Plateau. Observations of *R_s_* are impacted by the performance of instruments (pyrheliometer and pyranometers). These instruments should be accurately and regularly calibrated. Otherwise, they would lose their sensitivity and produce a spurious dimming trend of *R_s_*. On the contrary, the measurements of SunDu are much less sensitive to instrument calibration, as its recording material is replaced each day. There is significant evidence that the instruments used to measure *R_s_* at these four stations had important calibration problems before 1992 when the new instruments were deployed. The figure was produced using MATLAB.

To provide accurate decadal variability of *R_s_*, it is necessary to regularly and properly calibrate the instruments (i.e., the pyranometers) to measure *R_s_*. However, a world-wide radiometric reference for such a calibration was not established until the year 1979^18^,^19^. Without an accurate and regular calibration, the instruments would lose their sensitivity and introduce a false dimming trend of the observed *R_s_*^3^. SunDu recorders do not have this issue^20^ as their recording material, the light-sensitive paper, is replaced every day. Fig. 4 shows that there were obvious instrument degradation problems for the observed *R_s_* at the four stations in the Tibetan Plateau, while the SunDu-derived *R_s_* were more stable and more homogeneous.

**Figure 5:**
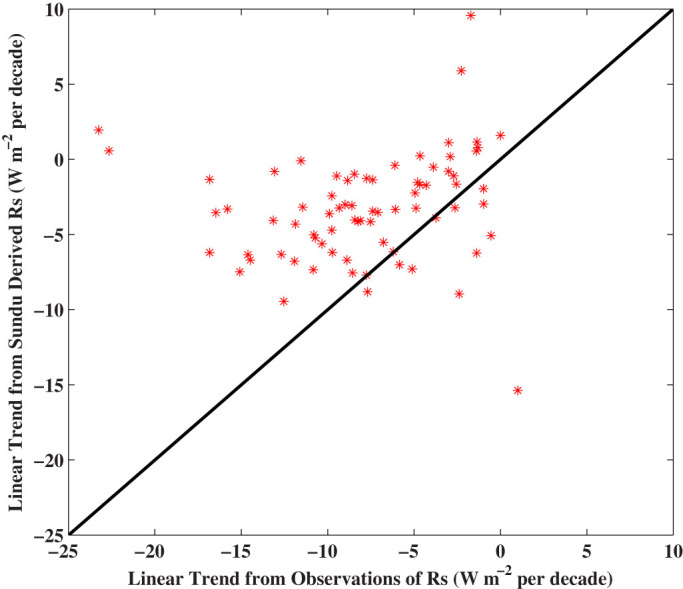
Scatterplot of the linear trends of observed *R_s_* as a function of trends of SunDu-derived *R_s_*. Each red star represents a station in China where the data duration of both the observed and calculated *R_s_* were longer than 120 months during 1960 to 1989. There are 76 stations in total. Linear trends of observed surface incident solar radiation (*R_s_*) are significantly more negative than those from sunshine duration (SunDu) derived *R_s_*. The figure was produced using MATLAB.

**Figure 6:**
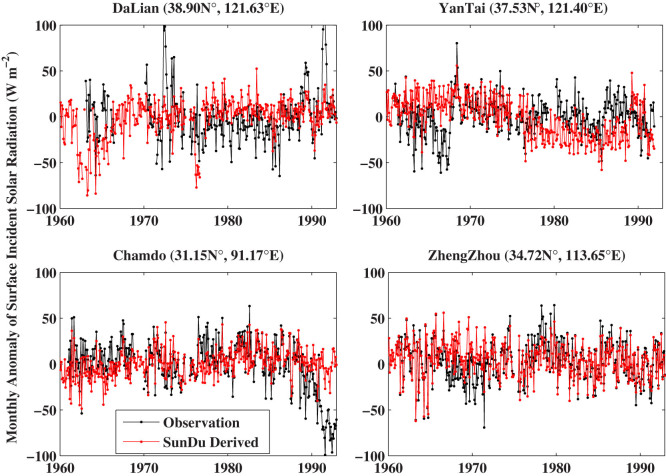
Time series of observed surface incident solar radiation (*R_s_*) and *R_s_* calculated from Sunshine Duration (SunDu) at four stations where the observed *R_s_* showed a near-zero trend while SunDu-derived *R_s_* had significant positive or negative trends (see Fig. 5). It is obvious that the instruments used to measure *R_s_* were inaccurately calibrated at the DaLian station in the early 1970s and the early 1990s, YanTai station in the late 1960s, and ChamDo station in the late 1980s to the early 1990s. At these stations, the trends of SunDu-derived *R_s_* are more reliable than those of observed *R_s_*. The figure was produced using MATLAB.

These instrument degradation issues were very common for the *R_s_* measurements in China before 1990. Fig. 5 shows that trends of *R_s_* from observations are substantially more negative than those of the SunDu-derived *R_s_* from 1960 to 1989. There are four stations where the observed *R_s_* had a near-zero trend while SunDu-derived *R_s_* had significant positive or negative trends. Fig. 6 shows that this is because the instruments used to measure *R_s_* were inaccurately calibrated at the stations at certain years.

**Figure 7:**
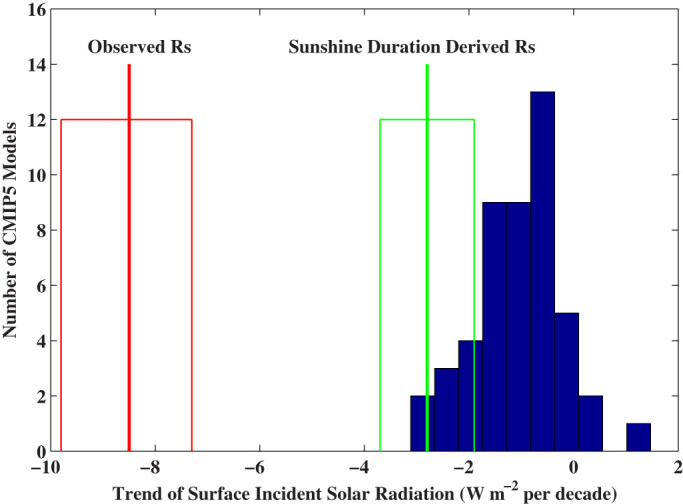
Histogram of the linear trends of surface incident solar radiation (*R_s_*, blue bars) over China from 1960 to 1989 simulated by 48 CMIP5 models (see Ma et al.^21^ for details of these models). None of the models reproduced the observed trend of −8.5 W m^−2^ per decade (thick red line, with the red box showing its 95% confidence interval) based on the raw data of *R_s_* observations. However, some CMIP5 models, including NASA Goddard Institute for Space Studies (GISS) models, National Center for Atmospheric Research (NCAR) Community Earth System Models (CESM-CAM5), and Centre National de Recherches Meteorologiques Climate Model 5 (CNRM-CM5), reproduced our new estimates of the trend based on Sunshine Duration (SunDu) derived *R_s_*, −2.8 W m^−2^ per decade (thick green line, with the green box showing its 95% confidence interval). The figure was produced using MATLAB.

Figs. 4–6 indicate that the lack of calibration or inaccurate calibration made the observed *R_s_* values highly uncertain. Fig. 7 further shows that measurement biases may explain the discrepancies between the observed and simulated decadal variability of *R_s_*. The linear trend of the observed *R_s_* averaged over China from 1960 to 1989 was −8.5 W m^−2^ per decade, with a 95% confidence interval from −9.8 W m^−2^ per decade to −7.3 W m^−2^ per decade. None of the 48 Earth System Models of CMIP5 (CMIP5 ESM)^7^,^21^ reproduced the observed trend (Fig. 7).

However, many high-quality CMIP5 ESMs reproduced the trend of SunDu-derived *R_s_* very well. The linear trend of the SunDu-derived *R_s_* averaged over China during the same period was −2.8 W m^−2^ per decade, with a 95% confidence interval from −3.7 W m^−2^ per decade to −1.9 W m^−2^ per decade (Fig. 7). The *R_s_* from NASA Goddard Institute for Space Studies (GISS) models show the best agreement with the SunDu-derived values. The four versions of GISS ESM simulated an average trend of *R_s_* by −2.8 W m^−2^ per decade. Other CMIP5 ESMs, including the Centre National de Recherches Meteorologiques Climate Model 5 (CNRM-CM5-2, −2.9 W m^−2^ per decade) and National Center for Atmospheric Research (NCAR) Community Earth System Models (CESM1-CAM5-1-FV2, −2.4 W m^−2^ per decade and CESM1-CAM5, −2.0 W m^−2^ per decade), also had consistent simulations of *R_s_* trend with SunDu-derived *R_s_*. However, the interannual variability of *R_s_* of CMIP5 is much less than those from the observed *R_s_* and SunDu derived *R_s_* because the models have difficulty in simulating variability of clouds^22^.

It has been shown that aerosol is the dominating factor for the dimming of *R_s_* in China from 1960 to 1990^23^,^24^ although cloud has been believed to be the key factor for the decadal variability of *R_s_* in the U.S.^25^,^26^ and India^27^ during recent decades. A recent study has shown that the CMIP5 models perform significantly different in simulating atmospheric aerosols over India^28^. This is because the atmospheric aerosol loading was interactively calculated by each individual model^10^, although the same emission inventory^29^,^30^,^31^,^32^ was used for all of the CMIP5 models^7^. Our results show that the GISS models perform best, which is partly because the GISS model team has made great effort in simulating aerosols^33^,^34^,^35^,^36^.

**Figure 8:**
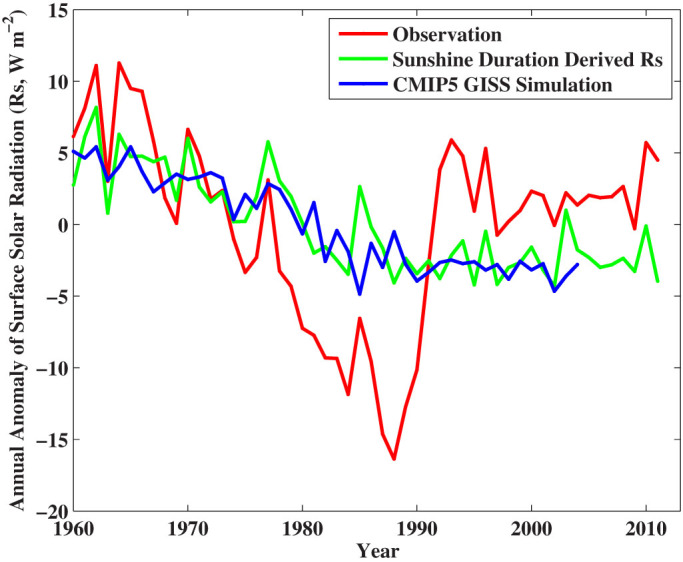
Time series of observed surface incident solar radiation (*R_s_*, in red), *R_s_* calculated from Sunshine Duration (SunDu, in green) and *R_s_* simulated by CMIP5 GISS (in blue) averaged over China. The observed *R_s_* overestimated the dimming trend from 1960 to 1989. The instrument replacement in China from 1990 to 1993 resulted in an abrupt increase to the observed *R_s_*. The agreement of the long-term trends of *R_s_* between sunshine duration (SunDu) derived *R_s_* and CMIP5 NASA Goddard Institute for Space Studies (GISS) model simulations (averaged from four different model versions) is much better than those between observations and CMIP5 simulations. After 1995, variability of the three estimates of *R_s_* from the observations, the SunDu and the CMIP5 model simulations agreed well because issues of the instruments used to measure *R_s_* had been eliminated. The figure was produced using MATLAB.

Fig. 8 shows that the agreement of the long-term trends between SunDu-derived *R_s_* and CMIP5 simulations is significantly better than those between observations and CMIP5 simulations over China from 1960 to 1989. The brightening rate of the measured *R_s_* was impaired by instrument replacement between 1990 and 1993^13^, as shown in Fig. 4. The observations of diurnal temperature range^8^,^37^ confirmed that the observed brightening from 1990 to 1993 was not real. The observed increase in *R_s_* is inconsistent with the observed increase in stratospheric aerosols because of Pinatubo volcano eruption in 1991^38^ either. After 1995, variability of the three estimates of *R_s_* from the observations, the SunDu and the CMIP5 model simulations agreed well because issues of the instruments used to measure *R_s_* had been eliminated. During this period, the impact of increased aerosols on *R_s_*^39^ has been offset by the decreasing cloud cover fraction^13^, and in turn *R_s_* kept near constant.

## Discussion

The overestimation of the dimming trend of the observed *R_s_* over China has been corroborated by independent studies on clouds and aerosols. Observed *R_s_* was reported to have decreased by 21 W m^−2^ in eastern China from 1961 to 1990^40^, with changes in aerosols regarded as the primary reason for this dimming trend^13^,^41^. Changes in cloud cover showed a decreasing trend^41^, implying an increasing trend of *R_s_*. The aerosol optical depth (AOD, vertical integration of optical extinction) over the same region during the same time has increased by 0.16^42^. Analyses have shown that *R_s_* under clear sky conditions decreases by 8 Wm^−2^ in the winter seasons or 9 Wm^−2^ in the summer seasons for an AOD increase of 0.1 over China^43^. The change in AOD can explain at most a reduction of 1.6 × 9 = 14.4 Wm^−2^ of *R_s_*, which is approximately two-thirds of the observed value, but probably considerably less because of masking by clouds. This is confirmed by the SunDu-derived *R_s_*, which showed a decrease of 8.4 W m^−2^ during the period.

Globally distributed *DTR* observations show that since 1985, brightening was only significant over Europe and there was a lack of brightening over other continents^8^. Furthermore, AOD derived from visibility observations has increased globally since 1973 except for Europe^39^. This explains why the CMIP5 ESM could only reproduce the observed brightening over Europe^5^.

The inhomogeneity of observed *R_s_* caused by inaccurate instrument calibration also impacted the observed *R_s_* over Europe before 1990. A homogeneity test was applied to *R_s_* measurements at 56 stations over Europe where at least 30 years of data of *R_s_* were available at each station^44^. Sixteen of the 56 series (28.6% of the total) were found to be inhomogeneous^44^. After these datasets were appropriately adjusted, the dimming trend over Europe from 1961 to 1984 became −2.0 W m^−2^ per decade, substantially less than the −3.1 W m^−2^ per decade derived from raw data of the observed *R_s_*^45^.

## Methods

Sunshine Duration (SunDu) records the time during a day that the direct solar beam irradiance exceeds 120 Wm^−2^. It was initiated 150 years ago and is one of the oldest and most robust measurements related to radiation^3^. Measurement of SunDu is insensitive to instrument calibration as its recording material is replaced each day.

SunDu has long been used to estimate *R_s_*, and the earliest and most popular methods are those developed by Ångström^46^ and subsequently modified by Prescott^47^, which assumed a linear relationship between relative *R_s_* and SunDu. Yang et al. proposed a revised Ångström-Prescott to estimate daily mean *R_s_* from SunDu^11^: 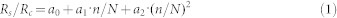
where n is the measured SunDu; N is the theoretical values of SunDu, and *R_c_* is the daily solar radiation at the surface under clear-sky conditions: 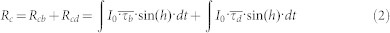
where *R_cb_* is the daily mean direct solar radiation at the surface, *R_cd_* is the daily mean diffuse solar radiation at the surface, *I*_0_ is the solar radiation at the extraterrestrial level, 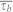 is the atmospheric transmittance for direct solar radiation, 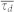 is the atmospheric transmittance for diffuse solar radiation, *h*(rad) is the altitude angle of sun, and *t*(s) is the time. The atmospheric transmittances 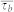 and 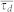 depend on Rayleigh scattering, aerosol extinction, ozone absorption, water vapor absorption and permanent gas absorption. Rayleigh scattering and water vapor absorption can be calculated from surface meteorological observations, and ozone and permanent gas absorption can be calculated using their climatological values. In the calculation of aerosol extinction, winter- and summer-averaged aerosols based on Hess et al.^48^ were included but the inter-annual variation of aerosols was not incorporated. Please refer to reference 11 for detailed information of the calculations.

The calculation of *R_c_* does not include time varying aerosols because SunDu is impacted by changes in both clouds and aerosols. Direct solar radiation is generally lower than 120 W m^−2^ for scattered clouds (cumulus, stratocumulus)^20^. High and thin cirrus, as well as aerosols can reduce SunDu at low solar elevations, i.e., at times when the incident clear sky solar radiation is not much larger than 120 W m^−2^. Recent studies confirmed such an inference and have shown that SunDu can accurately reflect the impact of change of aerosols and clouds on *R_s_*^12^,^13^,^14^,^15^,^16^,^17^ at time scales ranging from daily to decadal.

The parameters of Eq. (1), namely *a_0_*, *a_1_*, and *a_2_*, can be obtained by tuning this equation with measurements of *R_s_* and SunDu. In the existing studies, a suite of parameters are derived by calibrating Eq. (1) and then the method is applied regionally or globally^12^,^13^. This may limit the accuracy of the *R_s_* estimates. In contrast, in this study, we calibrate Eq. (1) at each station in China where both *R_s_* and SunDu observations were available at more than 10 years. We then apply Eq. (1) to calculate *R_s_* from 1958 to 2012 at each station when observations of SunDu were available. Figs. 2–3 show that *R_s_* can be calculated accurately from SunDu data at daily and monthly time scales. These two estimates of *R_s_* allow us to investigate the homogeneity of these two estimates, which are shown in Figs. 4–8. In this study, we show that the *R_s_* decreased at a rate of −2.8 W m^−2^ per decade from 1960 to 1989 over the 76 stations where both the observed *R_s_* and SunDu were available at more than 120 months during the study period. In a previous study, Tang et al.^12^ calculated *R_s_* from SunDu with a suite of parameters of Eq. (1) for 716 weather stations in China and estimated an averaged trend of −2.3 W m^−2^ per decade from 1961 to 2000, which is a little weaker than our current estimate because *R_s_* stopped decreasing after 1990 (Fig. 8).

In this study, we propose to combine the advantages of the observations of *R_s_* and SunDu. The observed *R_s_* can accurately quantify the variation of *R_s_* in higher temporal resolution, i.e., hourly, and daily. However, it is impaired by the sensitivity drift of its measurement instruments. This limits its usage in climatic study. The SunDu is nearly free from the sensitivity drift problem. We use the observed *R_s_* to calibrate Eq. (1) used to estimate *R_s_* from SunDu at each station. This makes up the disadvantages of SunDu: (1) SunDu does not directly provide an estimate of *R_s_*, (2) threshold of a SunDu recorder changes with recorder types and their sitting environment. In this paper, we show that the SunDu-derived *R_s_* has an advantage of long-term stability and can be used to climatic studies, i.e., to evaluate climate model simulations, which only require estimates of *R_s_* at coarse time resolution (monthly or annually). However, SunDu has difficulty in estimating *R_s_* at time scales shorter than daily.

## Notes

Reprints and permissions information is available online at www.nature.com/reprints.
